# Editorial: New advances in *Candida* infections: pathogenesis, diagnosis, and treatment

**DOI:** 10.3389/ffunb.2026.1869034

**Published:** 2026-05-21

**Authors:** Bruna Gonçalves, Sónia Silva

**Affiliations:** 1 Centre of Biological Engineering (CEB), University of Minho, Braga, Portugal; 2 LABBELS – Associate Laboratory, Braga/Guimarães, Portugal

**Keywords:** antifungal lock therapy, antifungal resistance, Candida spp, deep fungal infections, fungal biofilms, fungal virulence, vaginitis

## 1 Introduction

*Candida* species are opportunistic fungi that are responsible for a wide range of infections, from superficial mucosal candidiasis to life-threatening invasive candidiasis. The increasing prevalence of *Candida* infections, coupled with rising antifungal resistance and diagnostic challenges, underscores the critical need for a better understanding of their pathogenesis, more accurate and rapid diagnostic methods, and effective treatment strategies. Advances in molecular biology and immunology have begun to shed light on host-pathogen interactions, virulence factors, and resistance mechanisms. However, gaps remain in the early detection and management of resistant strains and the development of novel antifungal agents. Addressing these issues is vital for reducing the morbidity, mortality, and healthcare costs associated with *Candida* infections, especially among immunocompromised populations. The Research Topic “New Advances in *Candida* Infections: Pathogenesis, Diagnosis, and Treatment” features five contributions that collectively provide an overview of the current state of the field while also highlighting recent advances in understanding, diagnosing, and treating *Candida* infections. The aim of this editorial is to summarize the key themes addressed within the Research Topic, emphasize the scientific and clinical contributions of the included articles, and offer insights into potential future directions in the field.

## 2 Overview of the Research Topic

The contributions included in this Research Topic collectively illustrate how *Candida* species evolve and persist in clinical settings through environmental adaptation, drug resistance, and biofilm formation. These processes create significant challenges for diagnosis, treatment, and infection control. Within this conceptual framework, the contributions can be organized into two complementary domains: *i.) Candida* epidemiology, pathogenicity, and resistance *ii.*) *Candida* diagnostic approaches.

### 2.1 *Candida* epidemiology, pathogenicity, and resistance

Zhao et al. aimed to delineate the clinical characteristics, mycological profiles, and risk factors of deep fungal infections and to evaluate factors associated with mortality among inpatients in a general hospital in southwestern China. A total of 462 cases of deep fungal infections were identified (0.052% of hospitalizations) from 2020 to 2024. *Candida* remained the predominant pathogen (84.2%), while *Aspergillus* (13.9%) and *Cryptococcus* (6.71%) showed rising prevalence. The respiratory tract was the primary site of infection (88.6%). The majority of patients received azole therapy, although echinocandin treatment was associated with significantly higher survival rates. Key risk factors for poor prognosis include mixed infections, hematologic diseases, and the use of biological agents. Overall, the study highlights a sustained increase in deep fungal infections and an emerging threat from non-*Candida* pathogens, underscoring the need for improved prevention, diagnosis, and treatment strategies.

Two other articles focused on *Candida* pathogenicity and antifungal resistance. Kumari et al. highlighted how environmental iron availability regulates biofilm formation and the production of virulence-associated enzymes in emerging clinical *Candida* species, a finding that is relevant to both diagnostic and therapeutic strategies. Notably, *Candida utilis* exhibits iron-responsive increases in phospholipase and proteinase activities, accompanied by enhanced azole resistance. In contrast, *Candida auris* demonstrates iron-linked enhancement of echinocandin resistance alongside sustained expression of key virulence-associated enzymes.

Complementing these findings, a genomic and phenotypic analysis of azole-resistant *Candida parapsilosis* isolates from Portugal revealed the growing threat of antifungal resistance Papuc et al. A phylogenomic analysis found that the Portuguese isolates are more closely related to those from the United States and Germany than to isolates sequenced thus far from the neighboring country, Spain. Although three genetic clades of *C. parapsilosis* were identified, all fluconazole-resistant isolates clustered together. This suggests that the rise in resistance may be linked to the emergence or introduction of this specific lineage. Overall, this study offers valuable insights into fluconazole resistance mechanisms in Portuguese *C. parapsilosis* isolates and advances our understanding of increasing resistance in Southern Europe.

Shi et al. examined the complex management of fungal catheter-related bloodstream infections (CRBSIs) in hemodialysis patients, emphasizing the tension between guideline-directed care and real-world clinical constraints. Their review delved into the biology of *Candida* biofilms, highlighting the transition from yeast to hyphal forms and the production of a protective extracellular matrix (ECM), rich in β-1,3 glucan. This matrix was found to impair antifungal penetration, particularly azoles, despite apparent *in vitro* susceptibility. The presence of metabolically inactive persister cells further contributes to treatment resistance and relapse. The authors also noted an epidemiological shift toward *C. parapsilosis*, known for its affinity for indwelling devices. The review evaluated antifungal lock therapy (ALT) as a potential bridging strategy, comparing agents such as ethanol, taurolidine, amphotericin B, and echinocandins. Ultimately, while catheter removal remains the gold standard, individualized approaches that incorporate biofilm-targeted strategies are essential when removal is not immediately possible.

Collectively, these findings underscore the growing threat of invasive fungal infections, particularly those caused by *Candida*. Evolving resistance, environmental adaptability, and clonal spread are making these infections more difficult to manage and control.

### 2.2 *Candida* diagnostic approaches

Diagnostic approaches for vaginitis were re-examined from both historical and practical perspectives by Agoni. The author emphasized that, despite being a longstanding technique, wet mount microscopy remains a rapid, cost-effective, and clinically valuable diagnostic tool, particularly in resource-limited settings, highlighting the continued importance of foundational diagnostic skills alongside modern technologies. The author concluded that wet mount microscopy is being dismissed from the routine gynecological visit too soon, regardless of its utility for the diagnosis of vaginitis. Diagnostic limitations in fungal catheter-related bloodstream infections were also addressed by Shi et al., particularly the reduced reliability of differential time to positivity in fungal infections.

Collectively, these contributions highlight the challenges inherent in diagnosing *Candida* infections, particularly in cases of vaginitis and fungal catheter-related bloodstream infections.

## 3 Conclusions and future perspectives

The contributions to the Research Topic “New Advances in Candida Infections: Pathogenesis, Diagnosis, and Treatment” highlight the growing complexity of *Candida* infections, driven by rising incidence, antifungal resistance, biofilm formation, and evolving epidemiology. These studies show how environmental factors, such as iron availability, influence virulence and resistance, while clonal spread of resistant strains, especially *Candida* parapsilosis, complicates control efforts. Biofilm mechanisms, including the extracellular matrix and persister cells, are key drivers of treatment failure, particularly in device-related infections, for which management options are limited. Diagnostic gaps remain, from underused tools such as wet mount microscopy to unreliable methods for invasive infections. Future progress depends on biofilm-targeted therapies, better rapid diagnostics, genomic surveillance, and personalized, multidisciplinary approaches for high-risk patients.

